# Special Issue “Cell Division: A Focus on Molecular Mechanisms”

**DOI:** 10.3390/ijms27177626

**Published:** 2026-08-26

**Authors:** Antonino Colanzi, Stefano Sechi

**Affiliations:** 1 Institute of Endotypes in Oncology, Metabolism and Immunology “G. Salvatore” (IEOMI), National Research Council (CNR), Via P. Castellino 111, 80131 Naples, Italy; antonino.colanzi@cnr.it; 2 Institute of Molecular Biology and Pathology, Italian National Research Council (CNR-IBPM), 00185 Rome, Italy

## 1. Introduction

There are two types of eukaryotic cell division: mitosis and meiosis. Mitosis ensures somatic cell proliferation, generating two identical diploid daughter cells from a single parent cell. This process is vital for tissue growth, maintenance, and the repair of damaged cells. In contrast, meiosis is a specialized division restricted to germ cells that leads to the generation of haploid gametes (sperm and oocytes) and introduces essential genetic variation through homologous recombination.

The mechanisms of cell division are governed by complex molecular networks [1] and surveillance mechanisms [2] that not only maintain genomic stability but also ensure the precise partitioning and inheritance of cellular membranes and organelles, such as the endoplasmic reticulum, Golgi complex, and mitochondria [3].

Failures in cell division, which result in genomic instability and aberrant organelle partitioning and inheritance, are directly linked to human pathologies, including tumorigenesis, neurodegenerative diseases, and developmental disorders [1,4], and can compromise oocyte quality, leading to infertility or early embryonic arrest [5].

Consequently, a comprehensive understanding of the molecular mechanisms regulating cell division is fundamental for modern biomedical research. Elucidating these pathways is essential for the development of new diagnostic and therapeutic strategies for combatting both genetic and proliferative disorders [3].

## 2. An Overview of the Published Articles

This Special Issue presents a valuable collection of nine papers aimed at elucidating novel molecular mechanisms that control cell division, thus improving our understanding of the process.

In the following paragraphs, I will describe them briefly without going into detail to encourage the reader to delve deeper themselves.

Hadjisavva and Skourides (contribution 1): Spindle orientation (SO) is regulated by redundant cortical mechanosensing and shape-sensing mechanisms that ensure division-plane fidelity. Focal adhesion kinase (FAK) is essential for aligning the spindle with force vectors in cells undergoing metaphase rounding, whereas anisotropic cells utilize a FAK-independent process. This dual regulatory system allows cells to adapt their division planes to diverse physical and mechanical environments. Consequently, these mechanisms play a critical role in tissue morphogenesis and homeostasis by responding to both intrinsic and extrinsic cues.

Anglada et al. (contribution 2): This study introduces a cell synchronization strategy consisting of using the inhibitor RO3306 to enrich RPE-1 cell populations at specific mitotic stages. Unlike traditional methods, which use combinations of chemical inhibitors and interfere with normal mitotic progression, this block-and-release approach preserves mitotic integrity and cell viability without causing chromosomal non-disjunction. It provides a reliable framework for high-temporal-resolution analysis of molecular division mechanisms. This method is particularly valuable for investigating the processes that drive genomic instability in human cells.

Scarberry et al. (contribution 3): This investigation of p31Comet splice variants reveals that Variant 1, which contains an additional 32 N-terminal residues, exhibits reduced stability and MAD2-binding capacity relative to Variant 2. Consequently, Variant 1 is less effective at deactivating the spindle assembly checkpoint and promoting mitotic progression. Its unique and exclusive expression in the testes indicates a specialized, tissue-specific role in germ cell regulation. These findings demonstrate that the unique N-terminus of p31Comet is a key modulator of its mitotic activity.

Alonso-Ramos and Carballo (contribution 4): The nucleolus serves as a regulatory hub for the sequestering and timely release of the phosphatase Cdc14 during meiosis. Timely Cdc14 release is crucial for inactivating CDK activity and ensuring proper chromosome segregation between the two meiotic divisions. This review highlights how feedback circuits involving CDKs, the polo-like kinase Cdc5, and Cdc14 regulate meiotic recombination. Furthermore, Cdc14 activates the resolvase Yen1 to repair unresolved recombination intermediates in both mitosis and meiosis. This process highlights how phase-separated compartments integrate the feedback loops necessary for maintaining genetic stability and diversity.

Barda et al. (contribution 5): While MAD2L2 dimerization is critical for DNA repair, research utilizing CRISPR/Cas9 shows that it is dispensable for early mitotic regulation. The binding of CDH1 to MAD2L2 appears to actively prevent homodimerization, shifting the cellular balance toward monomeric interactions during division. This finding suggests that the equilibrium between monomers and dimeric forms is a fundamental factor in regulating MAD2L2-containing complexes. These results clarify the specific roles of MAD2L2 across different cellular processes.

Borseth et al. (contribution 6): The alignment of the sex trivalent (X1, X2, Y) was studied in the mantid Rhombodera megaera during male meiosis I. Quantitative imaging demonstrated that X1 and X2 kinetochores associate with fewer microtubules relative to the single, more robust Y kinetochore. This asymmetry in microtubule density balances the forces within the spindle, allowing the trivalent to align precisely at the equator alongside autosomes. These observations support the evolutionary significance of metaphase alignment for accurate chromosome segregation.

Wu et al. (contribution 7): Amyloid-beta (Aβ) peptides induce neurotoxicity in Alzheimer’s disease by triggering aberrant cell cycle reentry (CCR) in post-mitotic neurons. STAT3-dependent induction of PKCδ over-activates CDK5 via calpain2-mediated p35 cleavage, leading to caspase-3-mediated apoptosis. Inhibition of PKCδ was found to restore neuronal morphology and viability by mitigating the expression of G1, S, and G2 markers. This pathway provides a potential therapeutic target for mitigating Aβ-induced neuronal loss.

Piergentili and Sechi (contribution 8): Mitochondrially derived non-coding RNAs (mt-ncRNAs), including lncRNAs and circRNAs, are emerging as key regulators of gene expression and cellular homeostasis. Dysregulation of these transcripts is linked to failures in cell cycle control, resulting in growth defects and the promotion of apoptosis. Furthermore, specific mt-ncRNAs contribute to carcinogenesis by driving tumor hallmarks such as cell migration and metastasis. Understanding these interactions offers new perspectives for diagnostic and therapeutic applications in oncology.

Matsuura et al. (contribution 9): In *Drosophila* male meiosis, COPII proteins are essential for anchoring the contractile ring to the plasma membrane during cytokinesis. The COPII complex facilitates anterograde vesicle transport from the ER to the Golgi, supplying critical factors like DE-cadherin to the cleavage site. Depletion of COPII subunits leads to actomyosin ring detachment and the formation of multinucleated cells. These findings emphasize the importance of de novo membrane insertion for the completion of meiotic divisions.

We are grateful to all the authors who submitted their work and supported this collection, along with the *International Journal of Molecular Sciences* staff, whose help was invaluable to the success of this editorial project.
